# Comparison of cellular activity and gene expression in repaired versus reconstructed anterior cruciate ligaments: A case–control study

**DOI:** 10.1002/jeo2.70364

**Published:** 2025-07-21

**Authors:** Edoardo Monaco, Alessandro Annibaldi, Adnan Saithna, Fabio Marzilli, Alessandro Carrozzo, Vincenzo Visco, Andrea Ferretti, Danilo Ranieri

**Affiliations:** ^1^ Department of Orthopaedics Sant'Andrea Hospital, La Sapienza University of Rome Rome Italy; ^2^ AZBSC Orthopedics Scottsdale Arizona USA; ^3^ Santo Spirito Hospital Pescara Italy; ^4^ Department of Clinical and Molecular Medicine Sapienza University Rome Italy; ^5^ Institute for Sports Medicine and Science, Italian Olympic Committee Rome Italy; ^6^ Dipartimento di Scienze della Vita della Salute e delle Professioni Sanitarie, Università degli studi “Link Campus University” Rome Italy

**Keywords:** anterior cruciate ligament repair, anterior cruciate ligament reconstruction, cell biology, gene expression analysis

## Abstract

**Purpose:**

The aim of this study was to compare gene expression and cell biology in biopsy specimens from repaired and reconstructed anterior cruciate ligaments

**Methods:**

A biopsy of the repaired and reconstructed anterior cruciate ligaments was performed. Gene expression analysis of alpha smooth muscle alpha actin via immunofluorescence and gene expression analysis of Collagen I, Collagen III, Collagen I–III ratio, and p16 via reverse transcription polymerase chain reaction was performed. In addition, cell motility and migration were quantitatively assessed. Data analysis was performed using analysis of variance (ANOVA) and Tukey's multiple comparison tests.

**Results:**

Twenty‐five biopsies, 15 from repaired anterior cruciate ligaments and 10 from reconstructed anterior cruciate ligaments were obtained. Gene expression analysis showed a higher expression of Collagen I in the reconstruction group (*p* = 0.0027) while the expression of alpha smooth muscle actine, was significantly greater for the repair group (*p* = 0.001). Quantitative analysis demonstrated significantly higher cell motility in the cells from repaired ligament compared to cells cultured from grafts (43 vs. 54% residual open area from scratch, *p* = 0.0001 at 24 h; 10 vs. 17% residual open area from scratch, *p* = 0.001 at 48 h).

**Conclusions:**

Molecular analysis of repaired and reconstructed anterior cruciate ligaments demonstrates significant differences with respect to collagen quality, α‐SMA, and p16. dPCs cultured from biopsies tissue showed a greater cellular activation and migration capacity in the repaired ligament. The clinical importance of these findings is a confirmation that repaired anterior cruciate ligament tissue exhibits a cellular behaviour consistent with excellent biological healing potential.

**Level of Evidence:**

Level III, case–control study.

## Graphical abstract

A repaired anterior cruciate ligament show a cellular behaviour consistent with excellent biological healing potential.

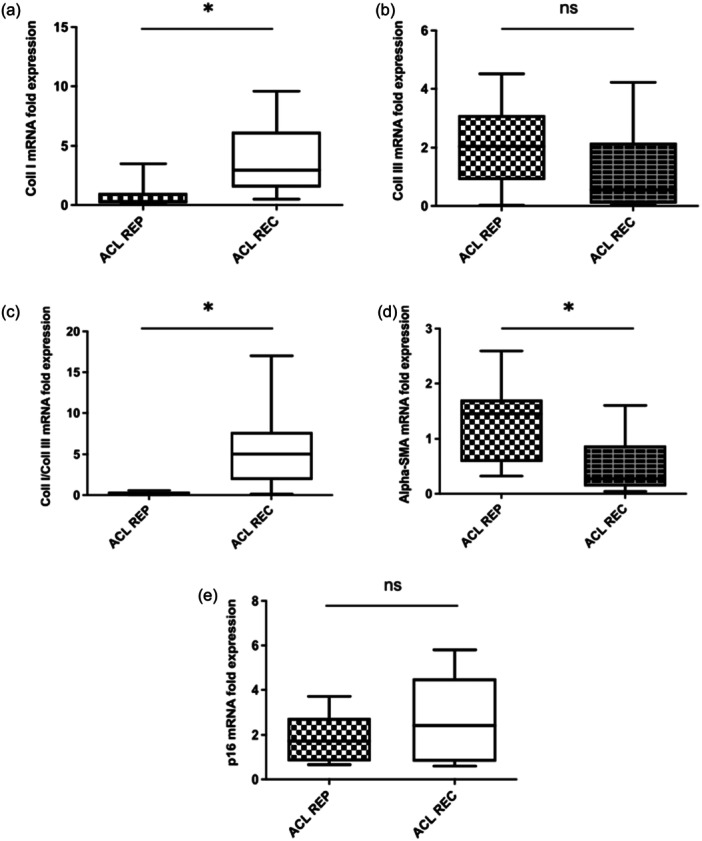

**Abbreviations**
ACL: anterior cruciate ligament
ACLR: anterior cruciate ligament reconstruction
ACLs: anterior cruciate ligaments
BMI: body mass index
CDKN2A/p16: Cyclin Dependent Kinase Inhibitor 2A/p16
Coll I: Collagen I
Coll III: Collagen III
Ct: cycle threshold
dPC: derived primary cultures
FBS: foetal bovine serum
MRI: magnetic resonance imaging
mRNA: messenger ribonucleic acid
PROMs: patient‐reported outcomes measures
RT‐PCR: reverse transcription polymerase chain reaction
α‐SMA: alpha smooth muscle actine

## INTRODUCTION

There has been a recent resurgence in interest in primary anterior cruciate ligament (ACL) repair, which was a popular treatment option for ACL tears in the 1970s and 1980s [2, 13]. Subsequently, ACL reconstruction (ACLR) became the gold standard because of high rates of failure observed at mid‐term follow up after ACL repair [8, 22]. The reason for the recent renewed interest in ACL repair is based on improvements in arthroscopic surgical techniques, suture materials, and rehabilitation protocols and contemporaneous reports of excellent clinical outcomes in selected patients [14, 17].

ACL repair offers a potential alternative surgical option to ACLR, especially in proximal tears. Theoretical advantages of ACL repair over reconstruction include a lack of donor site morbidity, shorter surgical time, better forgotten joint scores, quicker return of isokinetic muscle strength and return to sport, and non‐inferiority when compared to ACLR with respect to patient‐reported outcomes measures (PROMS), knee laxity parameters and graft rupture rates [1, 7, 8]. The reported advantages of ACL repair are attributed to the lack of donor site morbidity, lower overall surgical morbidity (avoidance of drilling large tunnels) and the preservation of the tibial footprint, proprioceptive fibres and biology of the torn stump [23]. These characteristics allow quicker rehabilitation (significantly better isokinetic muscle strength at 6 months post‐operatively), but potentially also better biological healing of the native tissue, which is not reliant on the maturation of a graft [4, 8].

The reparative response of the ACL is not fully understood and is unique in comparison to other ligaments of the knee [10, 11]. Historically, the ACL has been thought to have a poor healing ability compared to extra‐articular ligaments [10]. However, contemporary clinical and laboratory studies show that there is good healing potential [26]. Murray et al. [18] reported that the ruptured ACL goes through a four‐phase histologic process: an inflammatory phase, an epiligamentous phase where ACL stump retraction occurs, a proliferative phase and a remodelling phase. However, to the knowledge of the authors, molecular analysis and cellular activity of repaired ACL tissue in humans has not been characterised.

The aim of this study was to compare the cellular biology and gene expression of biopsied tissue from repaired and reconstructed anterior cruciate ligaments (ACLs). The hypothesis was that biopsies of repaired ACL tissue would demonstrate cellular biology favourable for healing when compared to ACL graft tissue and that there would be significant differences in gene expression between the groups.

## METHODS

### Ethical approval and study design

Institutional Review Board approval was granted for this study. (5665‐19). All patients gave their informed consent for inclusion before they participated in the study. The study was conducted in accordance with the Declaration of Helsinki. Consecutive patients presenting to the emergency department of Sant'Andrea University Hospital) between January 2019 and January 2022 with an acute knee injury (within 15 days of injury), with clinical and magnetic resonance imaging MRI evidence of a complete tear of the ACL were considered for study eligibility. Patients were excluded if they met one or more of the following criteria: inability to undergo surgery within 15 days of injury, age < 18 years old, history of ipsilateral knee surgery, injuries to additional knee ligaments that required surgical intervention (except for anterolateral ligament injuries), Kellgren Lawrence Grade III or IV change in any compartment, participation in sports at a professional level, or refusal to participate in the study. ACL repair was performed on patients who met the following intra‐operative criteria: proximal ACL tear (Sherman I and II) and good tissue quality of the ACL remnant [24]. Patients with tears that failed to meet these criteria underwent acute ACLR with four‐strand hamstring autografts.

### Surgical procedures

#### ACL repair

ACL repairs were performed using the technique described by Ferretti et al. [9] The repair was performed by looping two ultra‐high‐molecular‐weight polyethylene sutures (FiberWire, Arthrex Inc, Naples, FL, USA) through the ligament using a lasso‐loop knot‐tying configuration; subsequently, a 3.5 mm femoral tunnel was made with an outside‐in fashion and the sutures were pulled‐out, passed through a cortical Button (DogBone, Arthrex Inc, Naples, FL, USA) and tied off on the lateral femoral condyle.

#### ACL reconstruction

ACL reconstructions were performed with four‐strand hamstring autografts (semitendinosus and gracilis). Femoral tunnels were made in an outside‐in fashion, and the graft was fixed to femur with an adjustable‐loop cortical suspensory fixation implant (TightRope, Arthrex Inc, Naples, FL, USA) and with an interference screw (BioComposite, Arthrex Inc, Naples, FL, USA) on the tibial side.

### Tissue sampling

For the purposes of this study, part of the routine planned follow up involved an in‐office biopsy of the ACL at 8–12 months post‐operatively, in those patients who underwent repair. This was performed with a 1.9‐mm needle arthroscope (NanoScope, Arthrex Inc, Naples, FL, USA) under local anaesthesia. The sampling was performed at the proximal third of the repaired ligament through the anteromedial portal with the use of a 2 mm diameter biter (Nanobiter, Arthrex Inc, Naples, FL, USA). The sampled fragment had a volume of approximately 3 mm^3^. Enroled patients who had an ACLR during the study period and needed re‐operation (for any indication other than graft rupture), underwent graft biopsy in the same manner at the time of re‐operation. All patients who underwent re‐operation were clinically stable and had no symptoms of persistent instability.

### Primary culture isolation

All biopsies were used for both molecular analysis and primary culture. Primary cultures were established using the following process. Tissue biopsies were cut into small pieces (0.5–1.0 mm^3^) and then digested with 2 mg/mL collagenase type I for 1 h at 37°C. The samples were centrifuged (1000 rpm for 10'), the supernatants discarded, and the pellet was cultured in a sterile flask, in DMEM medium, supplemented with 10% foetal bovine serum (FBS) plus antibiotics at 37°C and 5% CO_2_. Derived primary cultures (dPC) were expanded and, once they reached 80% confluence, at passage three, cells were detached with 0.5 mL of 0.05% trypsin/ethylenediaminetetraacetic acid, counted and then used for all experiments.

### Messenger ribonucleic acid (mRNA) evaluations

The expression pattern of the Collagen I (Coll I) and Collagen III (Coll III) genes combined with differentiation markers for alpha smooth muscle actine (α‐SMA), and senescence‐associated marker (p16) were used to compare the two tissue types (ACL repair and ACLR) [5, 6, 16].

### Evaluation of gene expression

The gene expressions of the following proteins were determined:

1. Coll I, Coll III, and the ratio (Coll I/Coll III); Coll I is the main fibrillar collagen found in tendons and provides the greatest tensile strength of mechanical forces. Coll III is found in granulation tissue formation in the early phase of wound healing and plays an important role in the regulation of tissue regeneration [15].
2. The α‐SMA plays a crucial role during the activation of stromal cells in the wound healing of the ACL through restore the physiological tension of the tissues via the contraction of the extracellular matrix [16].
3. Cyclin Dependent Kinase Inhibitor 2 A/p16 (CDKN2A/p16), cellular aging marker responsible for the inactivation of replication and formation of new cells (referred to hereafter as p16 [25].

### RNA extraction and cDNA synthesis

All the samples obtained during the procedures were brought immediately to cryogenic temperature (−80°C). Subsequently, the total RNAs were extracted in a single session to avoid freezing and thawing cycles. Prior to nucleic acid extraction, the tissue fragments were reduced to fragments of up to 0.5 mm^3^ with the scalpel for a total of 50–100 mg. Subsequently the samples were homogenised through a homogeniser (T10 basic ULTRA TURRAX, IKA, Germany) for 3 min at RT in 1 mL of TRIzol (Invitrogen, Waltham, MA, USA). RNA was extracted using the TRIzol according to manufacturer's instructions and eluted with 0.1% diethylpyrocarbonate (DEPC)‐treated water. Each sample was treated with DNAase I (Invitrogen, Waltham, MA, USA). Total RNA concentration was quantitated by spectrophotometry. 1 μg of total RNA was used for reverse transcription using iScriptTM cDNA synthesis kit (Bio‐Rad, USA) according to manufacturer's instructions.

### Primers

Oligonucleotide primers necessary for target genes and the housekeeping gene were chosen utilising the online tool Primer‐BLAST (Invitrogen, Waltham, MA, USA). Primer sequences were illustrated in Table 1. For each primer pair, a no‐template control and no‐reverse‐transcriptase control assays were performed, each of which produced weak signals.

**Table 1:** Primer sequences.

Primer	Sequence
COL1A forward	5′‐ACATGT TCAAGCTTTGTGGACCTCCG‐3′
COL1A reverse	5′‐ACGCAGGTGATTGGTGGGATGTCT‐3′
COL3A1 forward	5′‐AGGGTGTCAAGGGTGAAAGTGGGA‐3′
COL3A1 reverse	5′‐ACCAGCCAGACCAGGAAGACCC‐3′
α‐SMA forward	5′‐GCACCCCTGAACCCCAAG‐3′
α‐SMA reverse	5′‐ACGATGCCAGTTGTGCGT‐3′
CDKN2A/p16 forward	5′‐CGTGGACCTGGCTGAGGA‐3′
CDKN2A/p16 reverse	5′‐AATCGGGGATGTCTGAGGGA‐3′
18S forward	5′‐AACCAACCC GGTCAGCCCCT‐3′
18s reverse	5′‐TTCGAATGGGTCGTCGCCGC‐3′

Abbreviations: CDKN2A, cyclin dependent kinase inhibitor 2A; COL, collagen; α‐SMA, alpha‐smooth muscle actin.

### PCR amplification and real‐time quantitation

Reverse transcription polymerase chain reaction (RT‐PCR) was used to measure the gene expression of repair process‐related structural protein (Coll I and Coll III), activation/differentiation marker (α‐SMA) and senescence‐associated marker (p16). Real‐time RT‐PCR was performed using the iCycler Real‐Time Detection System (iQ5 Bio‐Rad, USA) with optimised polymerase chain reaction conditions. The reaction was carried out in a 96‐well plate using iQ SYBR Green Supermix (Bio‐Rad, USA) adding forward and reverse primers for each gene and one µl of diluted template cDNA to a final reaction volume of 15 µL. All assays included a negative control and were replicated three times. The thermal cycling programme was performed as described [21]. Real‐time quantitation was performed with the help of the iCycler IQ optical system software version 3.0a (Bio‐Rad, USA), according to the manufacturer's manual. Results are reported as mean ± standard deviation (SD) from three different experiments in triplicate.

### Scratch assay

Primary culture cells were seeded at 2 × 10^5^ cells on 30 mm plates and grown until confluence. Next, a standardised cell‐free area was introduced by scraping the monolayer with a sterile tip. After intensive washing, some plates were fixed with 4% paraformaldehyde for 30 minutes at RT and photographed immediately after scratching, representing a T0 control. The remaining plates were incubated for 24 h, 48 h and then fixed. The photographs were taken at 10X magnification using an Axiovert 200 inverted microscope (Zeiss) and the mean percentage of residual open area was quantitated by measuring the gap distance between the scratch edges (six different measures for each photograph) using ImageJ software. Then the mean percentage residual open area obtained was compared to the respective cell‐free surface in T0. The results are expressed as the mean values of three independent experiments ± SD. Student's t‐test was performed, and significance levels have been defined as *p* < 0.05.

### Immunofluorescence

dPCs, grown on coverslips, were fixed with 4% paraformaldehyde in phosphate‐buffered saline (PBS) for 30 min followed by treatment with 0.1 M glycine for 20 min and with 0.1% Triton X‐100 for additional 5 min at RT to allow permeabilization. Cells were then incubated for 1 h at 25°C with the following primary antibodies: α‐SMA (1:50 in PBS; Dako) monoclonal antibodies. The primary antibodies were visualised using goat anti‐mouse IgG‐Alexa Fluor 488 (1:200 in PBS; Life Technologies) antibodies for 30 min at RT. Nuclei were stained with DAPI (Sigma). Coverslips were finally mounted with mowiol (Sigma) for observation. Fluorescence images were taken using conventional fluorescence with an Axiovert 200 inverted microscope. Quantitative analysis of the percentage of α‐SMA positive cells was assessed, counting for each sample a total of 20 cells, randomly observed in 10 microscopic fields from three different experiments. Results are shown as means ± SD. Student's *t* test was performed, and significance levels have been defined as *p* < 0.05.

### Statistical analysis

Relative gene expression was assessed normalising the cycle threshold (Ct) of the target gene to ribosomal 18S RNA and fold increase was calculated using the 2^−ΔΔCt^ method [3].

The data was analysed using analysis of variance (ANOVA) to test for differences amongst all means. A Tukey's multiple comparison test was used to determine differences between selected groups. The significance levels were defined as *p*‐values ≤ 0.05. All the calculations were performed with the SPSS® Statistics software (version 27.0.1.0; IBM Corporation, Chicago, IL, USA) for MacOS.

## RESULTS

Twenty‐five samples were taken: a group of fifteen patients who underwent ACL repair and agreed to participate in the study and undergo planned post‐operative micro‐biopsy, and a group of ten patients who underwent ACLR and were re‐operated for non‐graft rupture related indications at a mean of 25.6 months (range 16–36 months), who also agreed to study participation and micro‐biopsy. The reasons for reintervention in the ACLR group were: secondary medial meniscectomy (*n* = 5); secondary lateral meniscectomy (*n* = 3) and intra‐articular loose bodies (*n* = 2) The demographic data of the two groups are reported in Table 2.

**Table 2:** Demographics.

	ACL repair (*n* = 15)	ACLR (*n* = 10)	*p* value
Age, years*	31.1 ± 12.9	44.5 ± 6.7	0.45
Sex (M; F), number	12; 6	10; 8	0.19
BMI, kg/m^2*^	20.6 ± 2.1	23 ± 2.6	0.23

Abbreviations: ACLR, anterior cruciate ligament reconstruction; BMI, Body Mass Index; F, female; M; male.

### mRNA results

A molecular approach was used to quantify any differences in gene expression between the groups.

**Figure 1:**
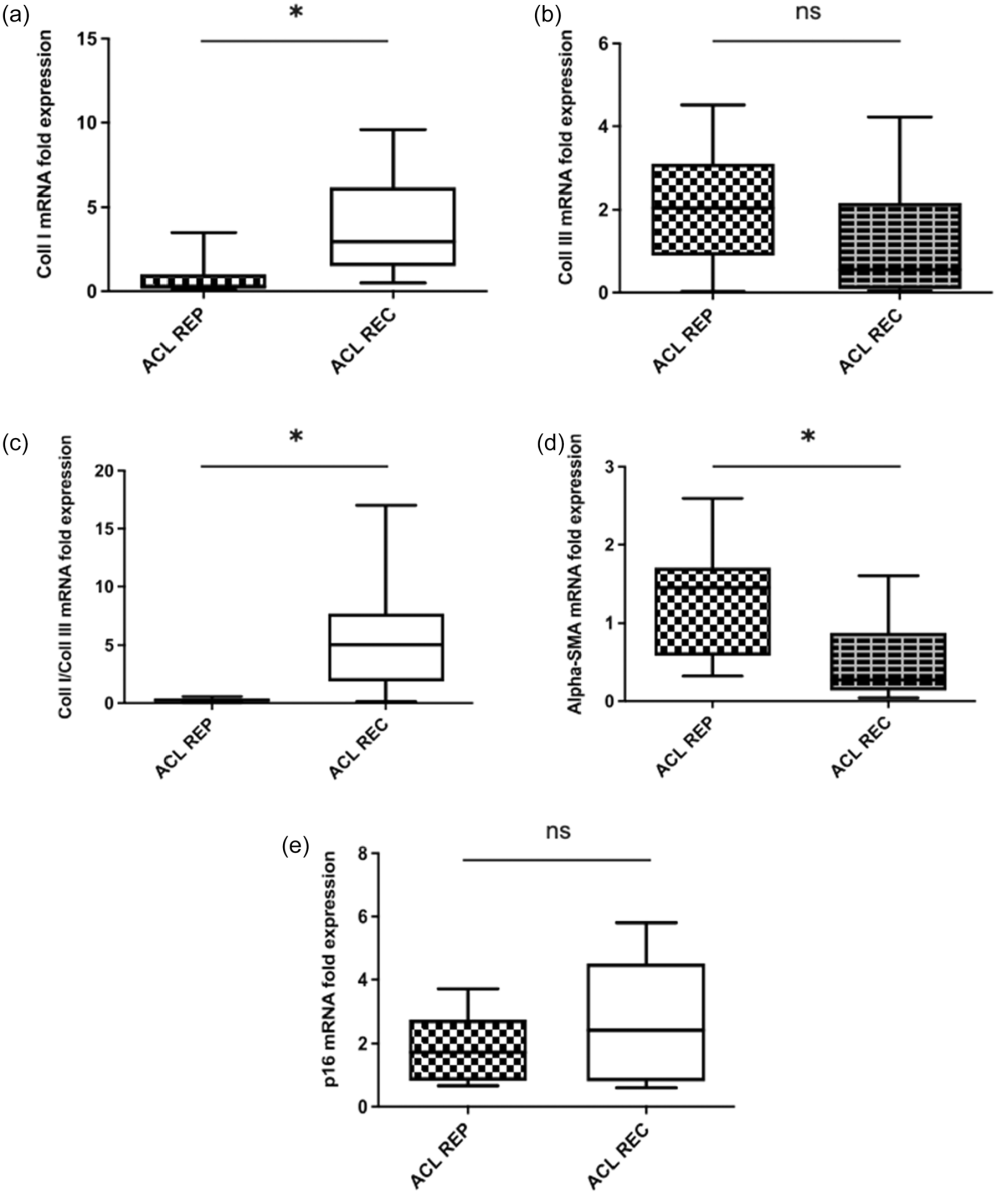
mRNA fold expression in the two groups (a–e). ACL REC, anterior cruciate ligament reconstruction; ACL REP, anterior cruciate ligament repair; Coll I, Collagen I; Coll III, Collagen III; α‐SMA, alpha‐smooth muscle actine expression.

mRNA expression patterns are represented in Figure 1a–e. A significant difference was observed among the two groups with greater expression of mRNA levels of Coll I gene in the ACLR group (*p* = 0.0027) (Figure 1a).

In the analysis of the Coll III gene expression pattern, a higher expression was found in the ACL repair group when compared to ACLR group, but these differences were not statistically significant (Figure 1b). Subsequently, the Coll I/Coll III ratio was higher (*p* = 0.0003) in the ACLR group when compared to the repair group (Figure 1c).

The mRNA expression levels of α‐SMA, as shown in Figure 1d, were significantly higher (*p* = 0.001) in the ACL repair group when compared to ACLR (Figure 1d).

The expression of p16 showed no statistically significant differences (Figure 1e).

### Immunofluorescence results

**Figure 2:**
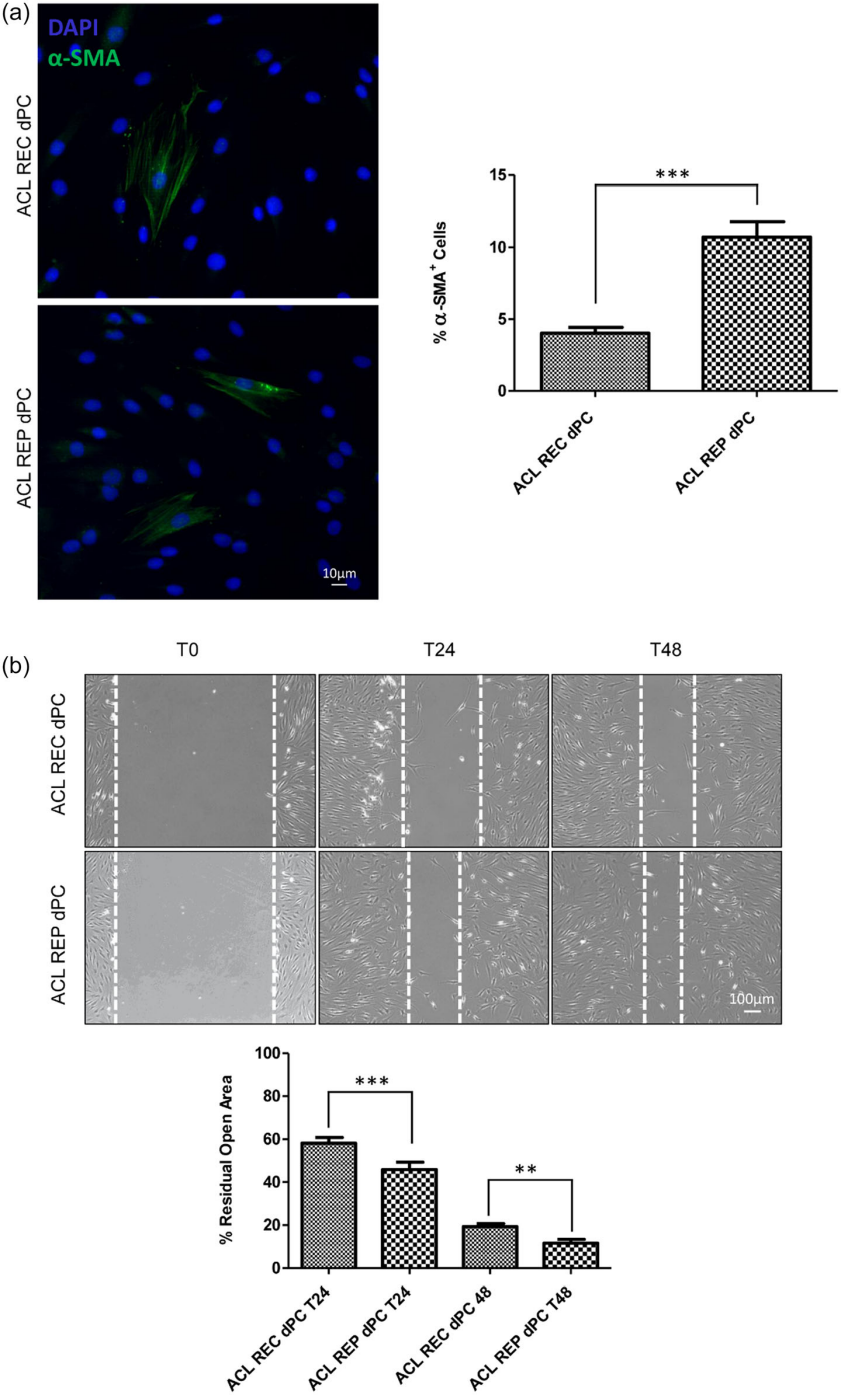
Expression of α‐SMA and migratory phenotype in ACL REC and ACL REP derived Primary Cultures. (a) The α‐SMA signal is present in intracellular filaments situated at the cytoplasmic periphery of cells. Nuclei are stained with DAPI. Bar 10 µm. Quantitative analysis of α‐SMA‐positive cells reveals a significant increase in ACL REP dPC. Student's t‐test was performed, and significance levels were defined as ***p < 0.0001. Values are expressed as mean ± standard deviation (SD) from three independent experiments. (b) Scratch assay on ACL REC dPC and ACL REP dPC after 24 and 48 h from scratch. The images are representative of three independent experiments. Results reported in the graph represent the mean percentage ± SD of a residual open area as described in material and methods. Results are shown as means percentage ± SD. Student's *t*‐test was performed, and significance levels have been defined as **p < 0.001 and ***p < 0.0001. Bar 100 μm. ACL REC dPC, anterior cruciate ligament reconstruction derived primary cultures; ACL REP dPC, anterior cruciate ligament repair derived primary cultures; DAPI, 4′,6‐diamidino‐2‐phenylindole; α‐SMA, alpha‐smooth muscle actine expression.

Expression of α‐SMA was assessed in ACLR and ACL repair dPCs by immunofluorescence which showed a localised signal in intracellular filaments organised in bundles. Quantitative analysis of α‐SMA‐positive cells revealed a higher (10% vs. 4% of α‐SMA + cells; *p* = 0.0001) α‐SMA positivity in ACL repair dPCs when compared to ACLR dPCs (Figure 2a).

### Scratch test results

The migration test for ACL repair dPCs demonstrated a typical migratory phenotype in most of the cells, and at both 24 and 48 h also showed a trend towards increased motility when compared with those of ACLR dPCs (Figure 2b). Quantitative analysis clearly indicated that the ACL repair dPCs cell motility was significantly higher compared to ACLR dPCs (43% vs. 54% residual open area from scratch, *p* = 0.0001 at 24 h; 10% vs. 17% residual open area from scratch, *p* = 0.001 at 48 h) (Figure 2b).

## DISCUSSION

The main finding of this study supported the hypothesis that ACL repair shows favourable healing characteristics when compared with reconstructed ligaments. Moreover, the comparison between ACLR and ACL repair dPCs showed a greater cellular activation and migration capacity in the repaired ligament which suggests that the repaired ACL shows greater biological healing potential.

The rationale behind the ACL repair performed in this study was to reattach the tibial remnant to femoral stump to counteract the factors that negatively affect ACL healing and ACL degeneration. Histological changes that occur after an ACL rupture have been well described in previous studies [10, 18]. The ACL undergoes rapid degeneration and resorption after an acute rupture due to the intra‐articular environment [10]. Murray et al. [18] reported that after injury, four phases proceed in the ACL: inflammatory phase within 3 weeks, epiligamentous phase between 3 and 8 weeks, proliferation and remodelling between 8 and 20 weeks. Murray et al also report that the response to rupture is comparable to that found in other dense connective tissues, with three differences: the development of a layer of synovial cells containing α‐SMA on the surface of the ruptured ends, the absence of tissue bridging the rupture site and the presence of an epiligamentous reparative phase which lasts from 8 to 12 weeks. However, they also showed that fibroblast proliferation, α‐SMA expression, and revascularization occurred also in the ruptured human ACL. According to the authors, this layer of synovial cells and the formation of epiligamentous tissue, a thin layer of connective tissue that covers the ligament and expresses the gene for the a‐SMA, plays a key role in the retraction and lack of potential regeneration of the ACL.

A study by Nguyen et al. [20] evaluated the reattachment of the tibial ACL remnant to the posterior cruciate ligament (PCL) during diagnostic arthroscopy before reconstruction. This phenomenon is a sign of the intrinsic healing response of the ACL, especially in proximal third tears. They further stated that the characteristics of ACL reattachment include solid attachment to the PCL, disorganisation of collagen fibres, increased neovascularization with abundance of myofibroblasts and high levels of type III collagen, as occurs in spontaneous healing processes of the MCL. However, healing of the ACL tibial remnant on the PCL typically results in a non‐functional ligament and knee instability. Regardless, they reported high α‐SMA levels in the reattached ACL. This supports the findings of the present study which show that the expression of α‐SMA in the ACL repair group is higher than the reconstructed group, so it is conceivable that the 'degeneration' of the ruptured ligament may result from its inability to approach the proximal stump. Furthermore, the increase of α‐SMA positive cells observed in ACL repair dPCs could indicate that differentiation into myofibroblasts plays a central role in tissue regeneration during the ligament repair process. In addition, they found high levels of Coll III in the reattached ligament as found in the present study in the repaired ligament.

Naraoka et al. [19] analysed gene expression of ruptured ACL cells during the four phases (inflammation, epiligamentous regeneration, proliferation and remodelling) to highlight histological changes in terms of cell characterisation. In their study, they noted the presence of Coll I and Coll III expression especially in Phase 1. This could potentially imply that an early ACL repair in proximal tears enhances the potential healing of the injured ACL. Our data regarding Coll I/Coll III, which shows comparable amounts of expression between a repaired and a healthy ACL, would therefore represent further evidence of the definitive healing of the treated ligament. Another major biological advantage of acute ACL repair is related to the presence of large amounts of multipotent mesenchymal cells, especially in Phase 1 and 2, which progressively decrease from Phase 3 onwards [16].

The low expression of p16 protein supports the hypothesis that the repaired ligament does not encounter an early degenerative response. The m‐RNA expression of Coll I and III, α‐SMA and p16 protein analyzed in the present study demonstrate that a repaired ACL heals in a favourable way, possibly thanks to mechanical stresses applied to the scar tissue because of the surgical repair [12]. Furthermore, the m‐RNA expression of a hamstring graft used to replace the ruptured ACL still differed from the repaired ACL in terms of Coll I/Coll III ratio, α‐SMA, and p16.

Limitations of this study include the analysis of gene expression only and not protein levels, cellularity or histological analysis of the scar tissue. However, this is because it is not feasible to safely harvest the required tissue volume to perform such analyses without risk of ACL rupture. For the same reason, multiple and repeated biopsies were not performed, and consequently the results of this study were limited to short‐term follow‐up only. Moreover, another limitation could be the different timing of sampling between the repair and reconstruction groups since the presence of secondary injuries unrelated to graft failure could alter the joint environment. In addition, the time between ACLR and reoperation was not considered, so it is not possible to know whether they were at the same phase of maturation. The small number of patients did not allow a comparison between molecular data and clinical outcomes. However, the molecular analysis seems to confirm the healing potential of the ACL. To the best of our knowledge, this is the first study to molecularly analyse a repaired ligament with in vivo sampling. The results suggested that an acutely treated proximal ACL injury has potential for healing. Although, this study is based on a molecular and cellular activity analysis and not on clinical outcomes, selective primary ACL repair represents a therapeutic approach to be considered in proximal ACL injuries in appropriately selected patients.

## CONCLUSIONS

Molecular analysis of repaired and reconstructed ACLs demonstrates significant differences with respect to collagen quality, α‐SMA and p16. dPCs cultured from biopsies of ACLR and ACL repair tissue showed a greater cellular activation and migration capacity in the repaired ligament. The clinical importance of these findings is a confirmation that repaired ACL tissue exhibits a cellular behaviour consistent with excellent biological healing potential.

## AUTHOR CONTRIBUTIONS

Edoardo Monaco, Danilo Ranieri, Vincenzo Visco and Andrea Ferretti have ideated the study and established the study design. Alessandro Annibaldi, Fabio Marzilli and Alessandro Carrozzo have collected the data. Danilo Ranieri and Fabio Marzilli performed the data analysis. Alessandro Annibaldi, Adnan Saithna and Alessandro Carrozzo draft and revise the manuscript. All authors read and approved the final manuscript.

## CONFLICT OF INTEREST STATEMENT

Edoardo Monaco and Andrea Ferretti are consultant for Arthrex. Other authors declare no conflicts of interest.

## ETHICS STATEMENT

IRB approval has been obtained for this study (5665‐19). Informed consent was obtained from all individual participants.

## Data Availability

Data are available from the corresponding author on reasonable request.
